# Comparing the Therapeutic Effects of Crocin, Escitalopram and Co-Administration of Escitalopram and Crocin on Learning and Memory in Rats with Stress-Induced Depression

**DOI:** 10.21315/mjms2021.28.4.6

**Published:** 2021-08-26

**Authors:** Mehran Joodaki, Maryam Radahmadi, Hojjatallah Alaei

**Affiliations:** Department of Physiology, School of Medicine, Isfahan University of Medical Sciences, Isfahan, Iran

**Keywords:** depression, stress, memory, learning, crocin, escitalopram

## Abstract

**Background:**

Depression affects various brain functions. According to previous studies, escitalopram influences brain functions in depression and crocin reduces memory impairments. Therefore, this study aimed to compare the therapeutic effects of using crocin and escitalopram (separately and in combination) on learning and memory in rats with stress-induced depression.

**Methods:**

Fifty-six rats were allocated into seven groups of control, sham, continuous depression, recovery period, daily injections of escitalopram, crocin and escitalopram-crocin during 14 days after inducing depression by stress. Passive avoidance (PA) test was used to assess brain functions.

**Results:**

Latency has significant differences in depression group. Also, it significantly increased in depression-crocin, depression-escitalopram and depression-escitalopram-crocin groups compared to the depression group. The dark stay (DS) time was significantly higher in the depression and depression-recovery groups. However, the DS time significantly decreased in the depression-crocin, depression-escitalopram and depression-escitalopram-crocin groups. Furthermore, the number of entrances to the dark room was significantly lower in depression-crocin and depression-escitalopram-crocin groups compared to the depression one.

**Conclusion:**

Different depression treatments (i.e. crocin, escitalopram and crocin-escitalopram) reduced depression-induced memory deficits. Crocin and escitalopram-crocin, respectively, improved brain functions and locomotor activity more than escitalopram. Comparatively, in subjects with depression, crocin, which is an effective saffron constituent, partially affected the memory deficits better than escitalopram (as a chemical component).

## Introduction

Depression is a heterogeneous disease triggered by social, biological, genetic and psychological (e.g. stressful life events) factors (1, 2). It affects many brain functions like cognition, memory, moods and other behaviours involving the limbic-cortical networks (3–6). This condition changes some neurobiological factors, including neurotransmitters (particularly serotonin), hormones, as well as inflammatory and neurotrophic factors in the brains of subjects with depression (5, 7–10). Nowadays, escitalopram or other selective serotonin reuptake inhibitors (SSRIs) are the first-line depression treatments (11). Nevertheless, the role of SSRIs on cognitive performance is not thoroughly clarified yet. The positive effects of SSRI drugs on cognitive functions among subjects with depression are previously reported in some studies (5, 11–13). Conversely, some research studies have indicated no improvement in memory due to using these drugs (14, 15).

Notably, some herbal drugs, such as saffron (*Crocus sativus*) play a definitive role in brain functions and cognitive capacity (16). Crocin is the most biologically active component of saffron (17) that can improve cognitive abilities, memory, anxiety and depression (17, 18). It can also improve the functionality of the central nervous system by regulating the serotonin synthesis (19). Even though both escitalopram and crocin affect serotonin secretion, they could probably have different mechanisms in affecting cognitive functions efficiently. Previous studies have focused on the effect of either crocin or escitalopram on providing treatment for depression; but no published report is yet available about the comparative therapeutic effects of crocin, escitalopram, co-administration of escitalopram and crocin, as well as the impact of allowing a recovery period after depression on cognitive functions. Therefore, this study aims to investigate which treatment (i.e. crocin, escitalopram, co-administration of escitalopram and crocin or recovery period after depression) would impact learning, memory, memory consolidation and locomotor activity in subjects with stress-induced depression (using behavioural tests).

## Methods

### Animals

**Figure 1:**
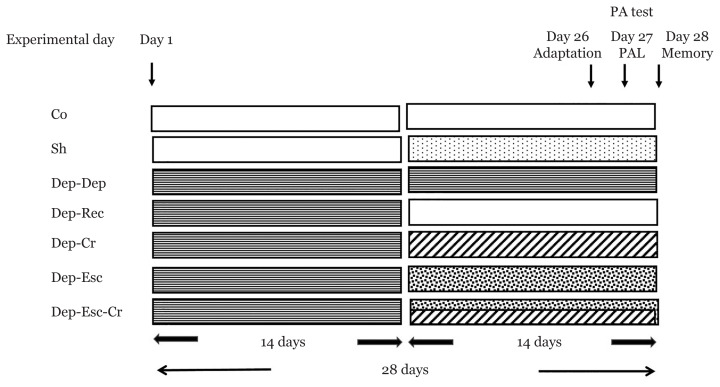
The schematic diagram of different groups

This experiment was performed using 56 adult male Wistar rats (with initial weights of 200 g–250 g). These rats were obtained from Isfahan University of Medical Sciences and were housed under controlled environmental conditions (23 ± 2 °C temperature and humidity of 50% ± 5% (mean ± standard deviation [SD]) with a 12:12 light/dark cycle (lights on from 07:00 to 19:00). Similar food and water were made accessible ad libitum except during the stress induction phase. Each colony group consisted of four rats in a cage. The experiment period lasted for 28 days. All behavioural tests were conducted between 14:00 and 16:00 daily. The Ethics Committee of Animal Use at the Isfahan University of Medical Sciences approved the study. Hence, all experiments were conducted in compliance with the Guide for the Care and Use of Laboratory Animals in Iran. After the adaptation of animals for a week, they were randomly divided into the following seven groups (*n* = 8):

- Control (Co) group: Rats were handled like the ones in the experimental groups during the study period and received no special treatments.
- Sham (Sh) group: Rats received equal volumes of normal saline (drug vehicle) consecutively for the next 14 days.
- Depression-recovery (Dep-Rec) group: Rats were under restraint stress (6 h daily for 14 days); subsequently, they were incaged for the next 14 days (recovery period).
- Depression-depression (Dep-Dep) group: Rats were under restraint stress (6 h daily, for 14 days) during two subsequent periods of the next 14 days (overall, 28 days).
- Depression-crocin (Dep-Cr) group: Rats were under chronic restraint stress (6 h daily, for 14 days); then they received daily injections of 30 mg/kg crocin for the next 14 days.
- Depression-escitalopram (Dep-Esc) group: Rats were under chronic restraint stress (6 h daily, for 14 days); then they received daily injections of 20 mg/kg escitalopram for the next 14 days.
- Depression-escitalopram-crocin (Dep-Esc-Cr) group: Rats were under chronic restraint stress (6 h daily, for 14 days); then they received daily injections of 20 mg/kg escitalopram and 30 mg/kg crocin for the next 14 days (Figure 1).

### Experimental Procedures

#### Depression paradigm

To induce depression, rats were placed in Plexiglas cylindrical restrainers for 6 h daily (8:00–14:00) during 14 days (20–22). On day 14, after verifying the induction of depression by a forced swim test (FST), the the rat with depression were introduced to the experimental protocol. An increase in immobility was seen as the induction of depression (23).

#### Chemicals and reagents

The subjects with depression received intraperitoneal injections of escitalopram (20 mg/kg; Sobhan Darou Pharmaceutical Co., Iran) and crocin (30 mg/kg; Sigma-Aldrich Co., USA), dissolved in normal saline for 14 consecutive days.

#### Behavioural paradigms

To assess learning, memory, memory consolidation and locomotor activity involving cognitive memory, the passive avoidance (PA) test was used. The PA apparatus (Shuttle-box, 64 cm × 25 cm × 35 cm) was divided into two identical compartments (32 cm × 25 cm × 35 cm) with a sliding door and grid floors. After a 300-sec habituation period in the apparatus (day 26), a single learning trial was performed (day 27) by delivering an electric shock through the grid floor (0.5 mA, 50 v and 2 sec) to the animal’s foot using an isolated stimulator. Subsequently, on day 28, the PA memory trial was conducted. Further details of the PA test were based on our previously published reports (18, 24). The initial latency time to enter the dark compartment was recorded before inducing the electric shock. In the memory trial on day 28, the latency to enter the dark room was measured up to a maximum delay of 300 sec. If the rat did not enter the dark compartment within 300 sec, the memory trial was terminated.

The difference between the initial latency and the latency after one day was interpreted as the occurrence of learning (25). Moreover, the latency of entrance to the dark compartment after one day was considered for measuring the short-term memory (26). The total dark stay (DS) time was assigned as either memory consolidation and/or storage of new information (25). Similarly, the number of entrances to the dark compartment was recorded as the locomotor activity (27, 28). Furthermore, the animal’s ability to remember the received foot shock was determined by the PA test. Avoidance to enter the dark compartment and even longer stay periods in the light compartment were interpreted as a positive response in the trial (25).

### Data analysis

All data were estimated as mean ± standard error of mean (SEM) and analysed by analysis of variance (ANOVA) test, followed by the Least Significant Difference (LSD) post-hoc testing for multiple groups. The paired-sample *t*-test was used to compare the initial latency and latency after one day (within-group). Additionally, the *P*-value less than 0.05 was considered statistically significant. All calculations were performed using IBM SPSS Statistics v.24.

## Results

In the current study, the Co and Sh groups exhibited no significant difference in the behavioural test. Therefore, the Co group was selected as a reference for all following comparisons.

### Assessment of Learning and Memory Functions

**Figure 2:**
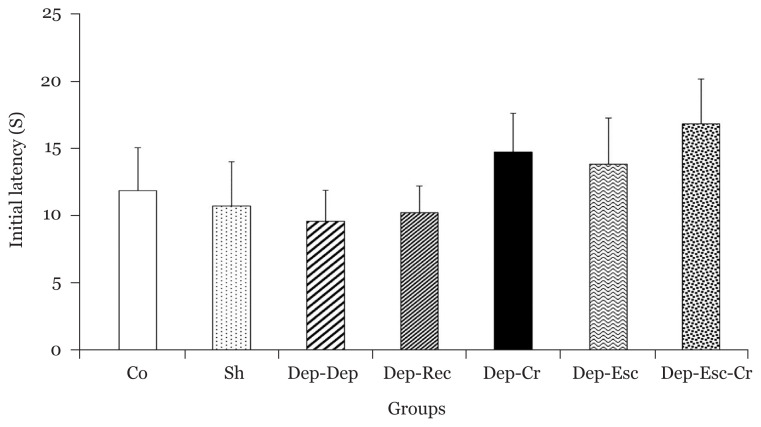
The initial latency to enter the dark room (of the PA apparatus) before receiving foot-shock for all groups (*n* = 8). Results are expressed as mean ± SEM. No significant differences were observed among all groups

**Figure 3:**
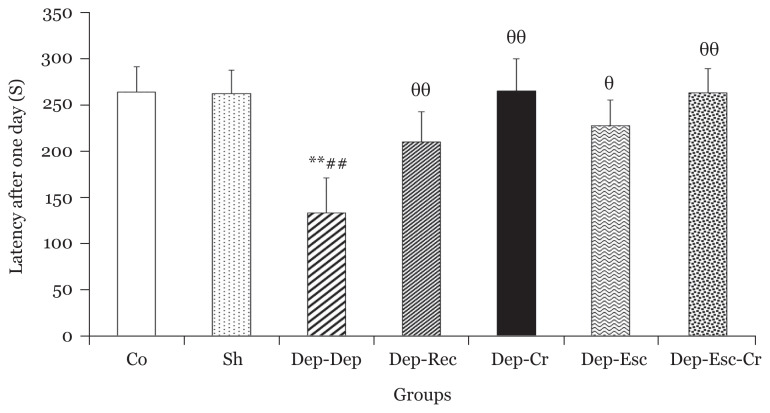
Latency to enter the dark room (of the PA apparatus), one day after receiving the foot shock for all groups (*n* = 8). Results are expressed as mean ± SEM Notes: ^**^P < 0.01 compared to the Co group; ^##^*P* < 0.01 compared to the Sh group; ^θ^*P* < 0.05 and ^θθ^*P* < 0.01 compared to the Dep-Dep group

As illustrated in Figure 2, no significant differences were observed in initial latency values among all groups. The latency after one day was significantly (*P* < 0.01) lower in the Dep-Dep group compared to the Co group; this indicated the induction of memory impairment by depression (Figure 3). Also, there was no significant difference in the latency after one day between the Dep-Rec and Co groups (Figure 3). However, the latency showed significant enhancements in the Dep-Cr and Dep-Esc-Cr groups (*P* < 0.01 for both) and the Dep-Esc group (*P* < 0.05) compared to the Dep-Dep group. Therefore, the beneficial effects of crocin and escitalopram treatments on reducing the depression-induced memory deficit was indicated (Figure 3).

**Figure 4:**
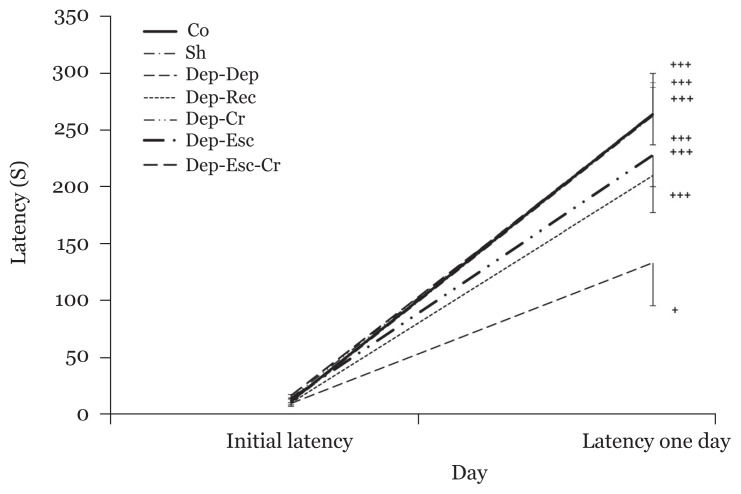
Initial latency and latency after one day to enter the dark room (of the PA apparatus), respectively, before and after the foot shock (within-group) (*n* = 8). Results are expressed as mean ± SEM Notes: ^+^*P* < 0.05 and ^+++^*P* < 0.001 Initial latency compared to latency after one day

The initial latency and latency after one day were analysed using a paired-sample *t*-test to evaluate within-group latency changes. Significant differences were detected between the initial latency and latency after one day in all groups (*P* < 0.001, except for the Dep-Dep group (*P* < 0.05), indicating the occurrence of learning in all experimental groups (Figure 4).

**Figure 5:**
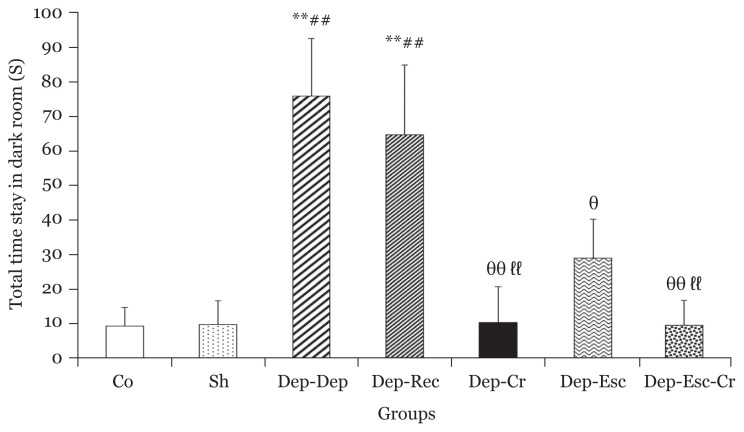
Total stay time in the dark room (of the PA apparatus), one day after receiving the foot-shock for all groups (*n* = 8). Results are expressed as mean ± SEM Notes: ^**^P < 0.01 compared to the Co group; ^##^*P* < 0.01 compared to the Sh group; ^θ^*P* < 0.05 and ^θθ^*P* < 0.01 compared to the Dep-Dep group; and finally, ^ℓℓ^*P* < 0.01 compared to the Dep-Rec group

In both Dep-Rec and Dep-Dep groups, the DS time was significantly (*P* < 0.01) higher than Co group, indicating the impairment of memory consolidation in depression with and without a recovery period. However, the total DS time had significant decreases in the Dep-Cr, Dep-Esc-Cr groups (*P* < 0.01) and Dep-Esc group (*P* < 0.05) compared to the Dep-Dep group (Figure 5).

**Figure 6:**
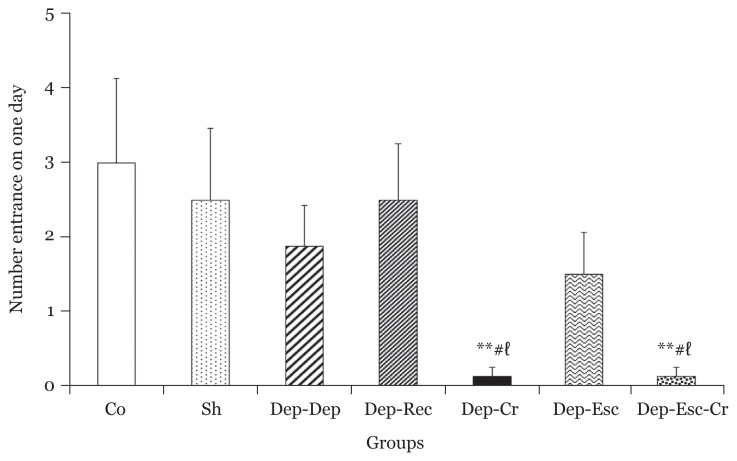
The number of entrances to the dark room in the PA apparatus for all groups one day after receiving the foot shock (*n* = 8). Results are expressed as mean ± SEM Notes: ^**^P < 0.01 compared to the Co group, ^#^*P* < 0.05 compared to the Sh group and ^ℓ^*P* < 0.05 compared to the Dep-Rec group

The number of entrances to the dark compartment showed no significant difference among the Dep-Rec, Dep-Dep and Dep-Esc groups (*P* > 0.05 for all groups) compared to the Co group (Figure 6).

However, there were significant decreases in the Dep-Cr group compared to the Co and Dep-Rec groups, respectively (*P* < 0.01 and *P* < 0.05). Similar enhancements were also observed in the Dep-Esc-Cr group compared to the Co and Dep-Rec groups, respectively (*P* < 0.01 and *P* < 0.05), indicating a reduced locomotor activity in both groups due to the use of crocin treatment in depression (Figure 6).

## Discussion

In this study, a comparison was conducted between the therapeutic effects of crocin, escitalopram, co-administration of escitalopram and crocin, as well as the recovery period after depression on cognitive functions (e.g. learning, memory, memory consolidation and locomotor activity) in rats with stress-induced depression.

Current findings revealed that learning occurred in all experimental groups although depression diminished the level of learning. As such, different treatments, such as the recovery period, crocin, escitalopram and escitalopram-crocin improved learning slightly among depression groups. According to some studies, depression often causes learning impairments (29, 30). Accordingly, Gao et al. (31) showed that learning decreased under the chronic unpredictable stress-induced depression models. Also, some reports have demonstrated that some materials, such as escitalopram and crocin may revert the learning impairments (32–36). Contrastingly, Skandali et al. (37) demonstrated that escitalopram not only did not improve learning, but also led to learning impairment among healthy volunteers.

In the present study, memory and memory consolidation were impaired by induction of depression. Consistent with these findings, depression accelerated the severity of cognitive impairments in other research studies (38–40). As Harmer et al. (41) suggested, the decrease in serotonin levels among depressed subjects caused impairment of memory consolidation.

Another important finding of the present study is that although the recovery period after depression slightly improved memory, it was not adequate to revert memory impairment among depressed subjects. Moreover, memory consolidation was severely destructed in subjects with depression (even when there was a recovery period).

According to other findings, different treatments, including the use of escitalopram, and particularly, crocin and crocin-escitalopram co-administration, improved memory consolidation and reduced depression-induced memory deficit. Moreover, the effects of escitalopram on other types of memory (12, 32) or the impact of citalopram (an older version of escitalopram) on memory consolidation are reported (41). However, Jensen et al. (42) have indicated that escitalopram did not revert those memory impairments that were induced by central 5-HT depletion (42). Also, in another study, escitalopram did not affect working memory (43). These contradictory results may have been due to the type of induced depression, strain, gender, drug dose and behavioural test (44). Nevertheless, there are pieces of evidence of memory improvement by crocin under various conditions (25, 45–47). Accordingly, Heidari et al. (48) showed that crocin could have a preventive effect on the memory impairments that were caused by age-related brain disorders, such as Alzheimer’s disease. Additionally, the beneficial impact of crocin on streptozotocin-induced memory impairment was seen to be related to antioxidant activities (49).

Interestingly, based on this study, even though using escitalopram and crocin separately improved memory impairments, the therapeutic effect of crocin, as an effective constituent of a medical plant, in the depression-induced memory deficits was stronger than the impact of escitalopram, which is a chemical component. Furthermore, the co-administration of crocin and escitalopram exerted more efficacy for the betterment of memory deficit in depression. As highlighted in a previous study, chronic stress would lead to the impairment of memory consolidation; whereas administration of crocin has improved it (25). Additionally, SSRIs have improved memory consolidation possibly due to the increase in serotonin levels (41). Different mechanisms were suggested for these beneficial effects of crocin and escitalopram on cognitive functions, namely: alteration of biochemical substances, brain neurotransmitters, hormones and the morphological changes in specific brain areas (32, 50–52). Both crocin and escitalopram are proven to have many similar beneficial effects. Nevertheless, the underlying mechanism of these effects with respect to providing treatments for memory impairments is not properly understood yet. For instance, it is demonstrated that both of these agents have antioxidant effects; hence, they both remove free radicals as one of the depression factors (53–55). Additionally, they prevent the elimination of neural cells by suppressing the effects of tumour necrosis factor (TNF) on neurons (56, 57). Moreover, they both increase serotonin in some brain regions (12, 19, 25) and reduce the hyperactivity of the hypothalamic-pituitary-adrenal (HPA) axis, which affects the hippocampal function and structure (58, 59). Therefore, further research is necessary to understand the differences between crocin and escitalopram. On one hand, it is proven that crocin increased hippocampal brain derived neurotrophic factor (BDNF) mRNA levels similar to some antidepressant drugs (17). On the other hand, in other studies, the antidepressant effects of escitalopram were unrelated to the regulation of hippocampal BDNF expression and serum BDNF levels among rats with depression (60, 61).

Other present findings revealed that depression decreased locomotor activity non-significantly in the PA test. However, Yang et al. (62) indicated that four weeks of unpredictable chronic stress (as a depression model) reduced locomotor activity in rats. Moreover, a decreased locomotor activity was observed by stress-induced depression using the open field test (63). By contrast, different evidence marked an increase in locomotor activity among mice with depression (64). Therefore, all factors, such as the type of behavioural test, rodent and stress, as well as duration and timing of stress-induced depression are important in various locomotor activity responses (62–64).

Finally, the use of crocin and co-administration of escitalopram and crocin, but not escitalopram alone, severely decreased locomotor activity compared to the normal conditions with a recovery period after the depression. It seems that crocin, but not escitalopram, had an effective role in reducing locomotor activity. Consistently with the present findings, Hosseinzadeh et al. (65) indicated that locomotor activity was reduced by crocin in rodents. Contrastingly, via an open field test, locomotor activity was not affected by crocin (66). There are limited studies on the effects of escitalopram on locomotor activity in rats with depression. Despite these findings, the level of locomotor activity in rats decreased in the post-stroke depression model while escitalopram reversed it (67). Also, Prinssen et al. (68) demonstrated an increase in locomotor activity by escitalopram. Thus, as previously mentioned, the level of responsiveness to locomotor activity may differ depending on the type of behavioural test and/or experimental protocol.

## Conclusion

All in all, different treatments for depression, including the use of escitalopram, crocin, and co-administration of escitalopram and crocin reduced the depression-induced memory deficits. Respectively, crocin and co-administration of escitalopram-crocin acted better than escitalopram alone on improving the brain functions and locomotor activity in depression. Concerning the alleviation of depression-induced memory deficits, crocin (as an effective constituent of saffron) seems to have partially acted better than escitalopram which is a chemical component. However, further studies on other molecular, cellular, structural and biochemical mechanisms are needed to assess the effects of escitalopram and crocin on improving brain dysfunctions both separately or in combination.
